# Metabolic defects in splenic B cell compartments from patients with liver cirrhosis

**DOI:** 10.1038/s41419-020-03060-1

**Published:** 2020-10-24

**Authors:** Man Huang, Xiaoju Liu, Haocheng Ye, Xin Zhao, Juanjuan Zhao, Yang Liu, Xiaomeng He, Mengmeng Qu, Jing Pan, Baidong Hou, Yongqian Cheng, Zhenwen Liu, Zhiwei Li, Lei Liu, Jian Sun, Shuye Zhang, Zheng Zhang

**Affiliations:** 1 Institute for Hepatology, National Clinical Research Center for Infectious Disease, Shenzhen Third People’s Hospital, 518112 Shenzhen, Guangdong China; 2 The Second Affiliated Hospital, School of Medicine, South University of Science and Technology, 518100 Shenzhen, China; 3 Institute of Biophysics, Chinese Academy of Sciences, 100101 Beijing, China; 4 Department of Infectious Diseases, Nanfang Hospital, Southern Medical University, 510515 Guangzhou, China; 5 State Key Laboratory of Organ Failure Research, Guangdong Provincial Key Laboratory of Viral Hepatitis Research, Nanfang Hospital, Southern Medical University, 510515 Guangzhou, China; 6 Research Center for Clinical & Translational Medicine, Fifth Medical Center for General Hospital of PLA, 100039 Beijing, China; 7 Department for Liver Transplantation, Fifth Medical Center for General Hospital of PLA, 100039 Beijing, China; 8 Department for Liver Transplantation, Shenzhen Third People’s Hospital, 518112 Shenzhen, Guangdong China; 9 Guangdong Key Lab of Emerging Infectious Diseases, Shenzhen Third People’s Hospital, 518112 Shenzhen, China; 10 Shanghai Public Health Clinical Center, Fudan University, 201508 Shanghai, China; 11 Institute of Hepatology, Shenzhen 3rd People’s Hospital, 5181112 Shenzhen, Guangdong Province China

**Keywords:** Immunological deficiency syndromes, Metabolic disorders

## Abstract

Liver cirrhosis is associated with defective vaccine responses and increased infections. Dysregulated B cell compartments in cirrhotic patients have been noticed but not well characterized, especially in the spleen. Here, we comprehensively investigated B cell perturbations from the spleens and peripheral blood of cirrhotic patients. We found that liver cirrhosis significantly depleted both switched and nonswitched splenic memory B cells, which was further confirmed histologically. Bulk RNA-seq revealed significant metabolic defects as the potential mechanism for the impaired splenic B cell functions. Functionally, the splenic memory B cells from cirrhotic patients showed strong metabolic defects and reduced proliferation compared with those from healthy controls. Thus, liver cirrhosis extensively disturbs the splenic and peripheral B cell compartments, which may contribute to defective humoral immunity during liver cirrhosis.

## Introduction

The human liver plays important roles in maintaining immune homeostasis^1^. During liver cirrhosis (LC), the immune functions of the liver are largely compromised^2^. Cirrhosis-associated immune dysfunction (CAID) underlies many pathological events, leading to severe complications and mortality in cirrhotic patients^3^. For example, in patients with advanced liver cirrhosis, immune deficiency generally contributes to increased susceptibility to microbial infections, frequently leading to patient death^4^. Alternatively, patients with compensated liver cirrhosis can suffer from acute-on-chronic liver failure (ACLF), which is initiated by various triggering events^5^. The onset of ACLF is related to CAID, during which imbalanced immunity perplexingly causes both hyperinflammation and immune paralysis, leading to dysregulated inflammatory injury during the triggering phase and failed immune defenses during the regenerative phase^5,6^. In addition, cirrhotic patients respond poorly to vaccination, rendering them the most vulnerable population to preventable infectious diseases, such as influenza^7^ and viral hepatitis^8,9^.

Humoral immunity plays crucial roles in protecting hosts from microbial infections by generating antibodies^10^. During the immune response, naïve B cells encounter antigens and follicular T helper (Tfh) cells in the secondary lymph nodes (SLOs), which triggers their proliferation and differentiation into CD27^+^ memory B cells and antibody-secreting cells^11^. Under steady state conditions, human memory B cells, plasma cells, and antibody pools cross regulate each other in a balanced manner^12^. However, liver cirrhosis triggers an imbalance in these regulatory mechanisms. Depletion of the circulating CD27^+^ memory B cell pool and simultaneous hyperglobulinemia in cirrhotic patients have been previously reported^13–15^. Interestingly, these phenomena have also been observed in various immune deficiency and autoimmune conditions^16–21^, suggesting possible common links underlying this imbalance.

Liver cirrhosis often causes increased spleen volume, and severe splenomegaly is treated through splenectomy^22^. The spleen is the largest SLO in the body and contains a large pool of immune cells, especially abundant B cells^23^. Although the spleen plays a key roles in immune defense against blood-borne infections^24^, the roles of the spleen in destroying immune cells or preventing harmful infections during liver cirrhosis are still under debate^25^. It is still unknown how CAID affects splenic immune cell compartments. Recently, we determined that an expanded hyperactive splenic Tfh cell population correlated with enhanced germinal center (GC) responses and hypergammaglobulinemia in cirrhotic patients^26^, showing the importance of investigating the regional immunity of the spleen in liver cirrhosis. Here, we comprehensively characterize the splenic B cell compartments from cirrhotic patients, investigate the underlying mechanisms of memory B cell depletion and functional impairment, and explore the consequences of these phenomena on the B cell repertoires and their potential clinical significance.

## Results

### The perturbations of peripheral and splenic B cell compartments in patients with liver cirrhosis

**Fig. 1:**
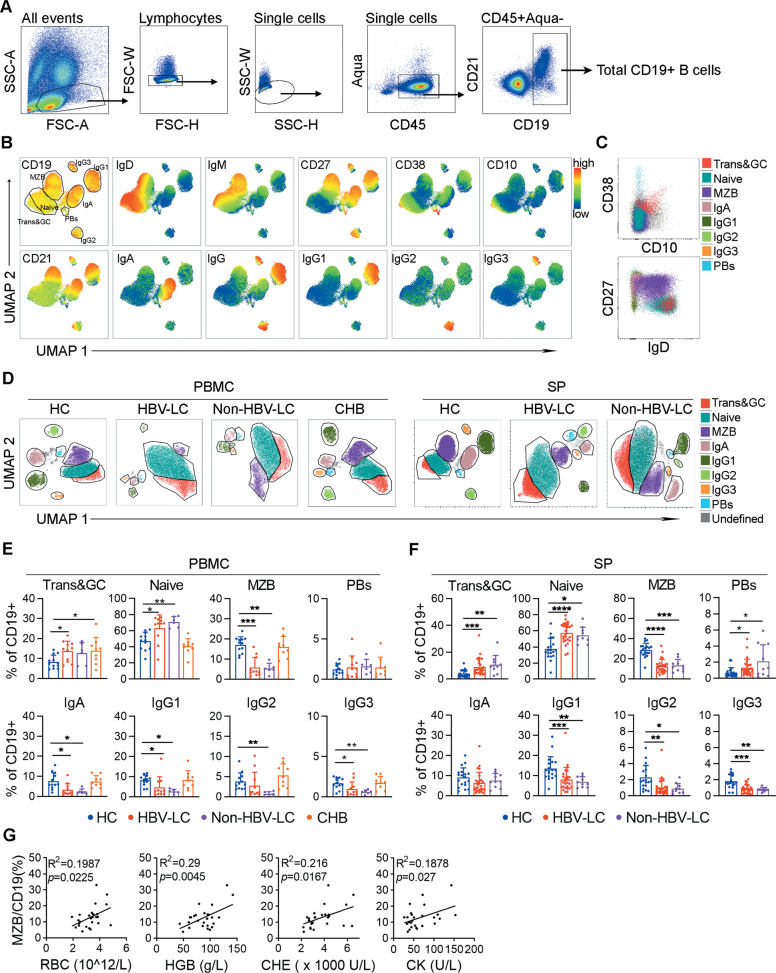
Perturbations of splenic and peripheral B cell compartments in patients with liver cirrhosis. **a** Gating strategy for total B cells from PBMCs and spleens by flow cytometry. **b** Expression patterns of the indicated markers on total splenic B cells gated as in **a** using UMAP in FlowJo software. The plot was from the data of one representative healthy donor. Eight subsets were gated as indicated by the UMAP heat map. **c** Overlay of the 8 B cell subsets with a unique color for each subset based on CD38 and CD10 expression and/or CD27 and IgD expression. **d** Representative subset gating of the B cells from the PBMCs of healthy donors (HC), HBV-LC, nonHBV-LC, and CHB subjects (left) and from the spleens of HC, HBV-LC, and nonHBV-LC subjects (right) based on UMAP. **e** Frequencies of the B cell subsets from the PBMCs of HC (*n* = 12), HBV-LC (*n* = 11), nonHBV-LC (*n* = 6), and CHB (*n* = 9) subjects. **f** Frequencies of the B cell subsets from the spleens of HC (*n* = 19), HBV-LC (*n* = 26) and nonHBV-LC (*n* = 10) subjects. *P < 0.05, **P < 0.01, ***P < 0.001, and ****P < 0.0001, as determined by Mann–Whitney *U*-test. **g** Correlation analysis of the splenic MZB frequencies and clinical markers, including red blood cells (RBCs), hemoglobin (HGB), cholinesterase (CHE) and creatine kinase (CK), in HBV-LC patients (*n* = 26). The Spearman rank correlation test was used, and R squared (*R*^2^) with *P* values are shown.

Although the peripheral CD27^+^ memory B cell pool is depleted in cirrhotic patients^13–15,27^, the splenic B cell compartments have not been well characterized. Here, spleen and peripheral blood samples were collected from cirrhotic patients (hepatitis B virus-associated cirrhosis (HBV-LC) and nonhepatitis B virus-associated cirrhosis (Non-HBV-LC)) and from healthy donors (Table 1) for a comprehensive splenic B cell study. Noncirrhotic patients with chronic hepatitis B (CHB) were also included in the peripheral B cell study for investigating the effect of HBV infection on B cells without cirrhotic conditions. The total splenic or peripheral B cell pools were investigated by a multicolor flow cytometric panel (Fig. 1a and Table 2, panel 1) that included CD45 and a group of B cell-related markers, such as CD19, CD21, CD27, CD10, CD38, IgD, IgM, IgA, IgG, IgG1, IgG2, and IgG3. The flow cytometric data were analyzed by FlowJo software with UMAP to reduce the high-dimensional parameters to a 2D map^28^. A total of eight subsets were identified based on UMAP clustering (Fig. 1b). Considering the similarities between the splenic and peripheral B cells, and considering the B cells subsets classifications by others^29–32^, these B cell subsets were named Trans&GC B cells (transitional or germinal center (GC) B cells, CD38^hi^CD10^+^), PBs (plasmablasts, CD38^hi^CD10^−^), MZB (marginal zone or marginal zone -like B cells, CD27^+^IgD^+^IgM^+^), naïve B cells (IgD^+^IgM^+^CD27^−^), and IgA/G1/G2/G3 memory B cells (IgA^+^, IgG1^+^, IgG2^+^ or IgG3^+^, and IgD^−^IgM^−^CD27^+/−^). The Trans&GC B cell and PB subsets differed from the other subsets due to their CD10 and CD38 expression, while the naïve, MZB and IgD^−^ memory B cells differed from the other subsets due to their IgD and CD27 expression patterns (Fig. 1c). Using this strategy, the frequencies of the total B cells and the B cell subsets from the spleen or periphery were analyzed, and compared between the patient groups and healthy controls. We found that the peripheral and splenic CD19^+^ B cell proportions were similar among the various groups (Fig. S1A). Importantly, both the peripheral blood and spleen from cirrhotic patients showed significant increases in Trans&GC and naïve B cells, and showed a dramatic decreases in MZB and IgG1, IgG2, and IgG3 memory B cells (Fig. 1d–f). In accordance with our previous study^26^, plasmablasts were found to be elevated in the spleens of cirrhotic patients. IgA memory B cells were found to be significantly decreased in peripheral blood; however, their counterpart in the spleen seemed to have largely remained at a level comparable to that of the healthy subjects (Fig. 1e, f). Notably, while the CHB patients had broad, normal B cell subsets similar to those of the healthy controls, their Trans&GC B cells did show an increase similar to that of the cirrhotic patients (Fig. 1e), indicating a common B cell signature in patients with liver injury.

**Table 1:** Basic demographic information of the enrolled subjects.

	HC	CHB	HBV-LC	Non-HBV-LC
Subjects	50	34	63	27
Spleen usage	27	0	44	11^#^
PBMC usage	27^▲^	34	24^▲^	16
Age (years)	45.7 (24–63)^*^	33.8 (17–56)	48.3 (26–66)	53.4 (23–78)
Gender (M/F)	38/12	26/8	46/17	7/20
Platelet (10^9^/L)	176.3 (5.5–440)	201.7 (69–377)	79.2 (9–404)	101 (25–233)
Albumin (g/L)	32.5 (15.6–49)	41.7 (27–48)	34.8 (23–48)	32.5 (22–42)
Tbil (µM/L)	17.1 (6.3–49.7)	31.5 (6.2–254.7)	18.2 (6.3–47.3)	28.7 (8.1–201.9)
DBil (µM/L)	8.5 (1.9–39)	20.5 (2.1–200.7)	8.2 (2.4–38.1)	16.3 (3.4–165.3)
ALT (U/L)	51.7 (9–476)	154.4 (8–969)	25.9 (7–136)	32 (11–86)
AST (U/L)	53.6 (13.1–283)	96.4 (15–697)	34.3 (15–174)	43.7 (14–109)
RBC (10^12^/L)	3.9 (2.2–6.3)	4.8 (4–5.7)	3.4 (1.7–5.5)	3.5 (2.2–5.3)
HGB (g/L)	112.6 (62–182)	144.7 (108–172)	95.2 (29–164)	101.7 (53–154)
CHE (1000 U/L)	5474.3 (2150–11257)	7847 (3984–13373)	4176.5 (1696–8838)	3960 (1435–6906)
CK (U/L)	453.8 (41–1787)	NA	66.1 (19–153)	90.4 (23–844)
HbeAg (+/−)	NA	27/7	10/53	0/27
Child-Pugh class (5/6/7/8)^$^	NA	NA	8/11/16/9	1/2/8/0

*M* male, *F* female, *TBil* total bilirubin, *DBil* direct bilirubin, *ALT* alanine aminotransferase, *AST* aspartate aminotransferase, *RBC* red blood cell, *HGB* hemoglobin, *CHE* cholinesterase, *CK* creatine kinase, *HBeAg* hepatitis Be antigen, *NA* not applicable.

^*^Clinical data of HC subjects were only from donors with spleen usage.

^#^The 11 nonHBV-LC patients included five PBC patients, two HCV-related patients, one alcoholic-related patient and three patients with cirrhosis of unknown reason.

^▲^Four HC and five HBV-LC PBMC samples were collected from four liver transplantation donors and five splenectomy patients, respectively.

^$^Child-Pugh class showed were from HBV-LC and Non-HBV-LC patients who underwent splenectomy to relieve the symptoms caused by portal hypertension and splenomegaly. The time of the clinical indexes collected for calculating Child-Pugh class were 1–2 weeks before the patients underwent splenectomy to avoid drugs affect.

**Table 2:** Antibody panels for flow cytometry.

Marker	Clone	Fluorochrome	Panels
CD45	2D1	APC-H7	Framework panel
CD19	SJ25C1	BUV496, BUV395, and BV711	
CD10	HI10a	BV605	
CD21	B-ly4	PE-Cy7/PE-CF594	
CD27	O323	BV785	
CD38	HIT2/HB7	BV711/PE-Cy7	
IgD	IA6-2	BV421	
IgM	MHM-88/G20-127	BV650/PE	
Aqua	(dead cell exclusion)	BV510	
IgG	G18-145	BUV395	Panel 1
IgA	IS118E10	PerCPVio700	
IgG1	HP6001	PE	
IgG2	HP6002	AF647	
IgG3	HP6050	AF488	
CD95	DX2	PE	Panel 2
FcRL4	413D12	PerCP-eFluor 710	
CD86	IT2.2	BV711/BV650	
CD11	S-HCL-3	APC	Panel 3
CXCR3	G025H7	PcrCP-Cy5.5	
CCR6	G034E3	PE	
CD71	M-A712	BV711	

The reduction in splenic MZB cells is a prominent feature in the perturbed B cell compartment in LC. The frequency of MZB cells in the spleens of cirrhotic patients did not correlate with their disease severity indexes, such as the Child-Pugh score, white blood cell counts and platelet counts (Fig. S1B, C). However, the frequency of MZB cells in the spleens did correlate with several clinical parameters, including cholinesterase (CHE), creatine kinase (CK), hemoglobin, and red blood cell (RBC) counts (Fig. 1d). These data suggest that liver cirrhosis disturbs the B cell compartment not only in the periphery but also dramatically in the spleen.

### The persistent activation of splenic B cell compartments in cirrhotic patients

**Fig. 2:**
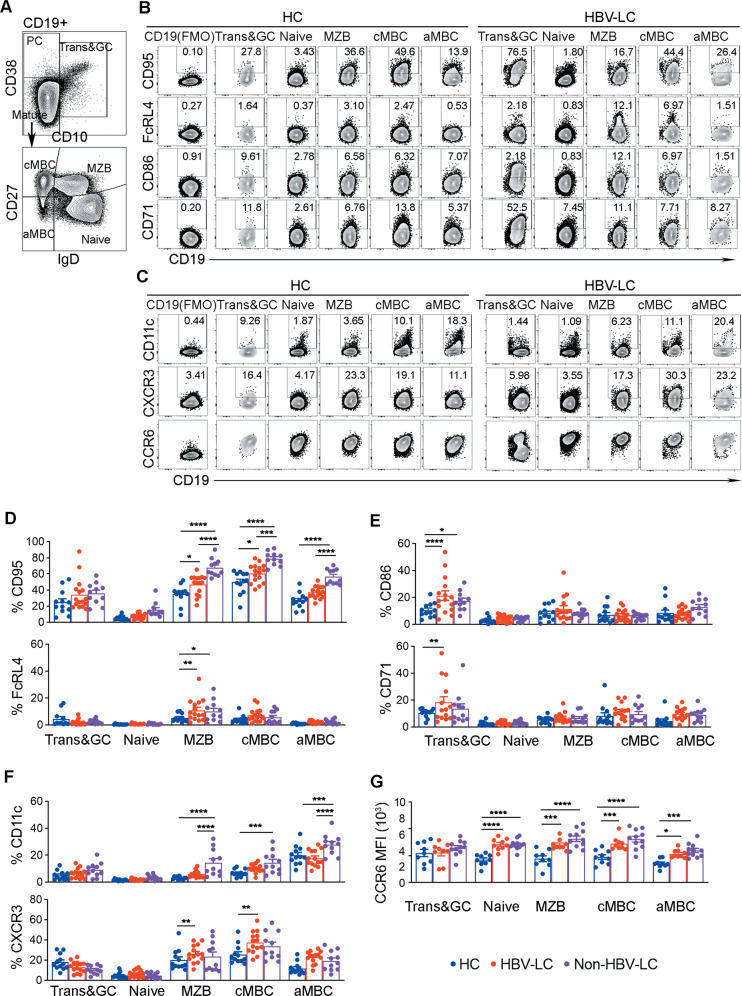
Phenotypical analysis indicates the persistent activation of the spleen B cell compartments in LC patients. **a** Gating of Trans&GC B cells, PBs, cMBCs, aMBCs, MZB cells, and naïve B cells on a FlowJo pseudocolor plot for subsequent phenotypic analysis. **b** Representative CD95, FcRL4, CD86, and CD71 expression profiles on the spleen B cell subsets from the spleens from one healthy donor and one HBV-LC donor. The positive populations were gated according to the Fluorescence Minus One (FMO) controls for each marker, and their percentages are indicated. **c**, **d** Percentages of the CD95^+^, FcRL4^+^, CD86^+^ and CD71^+^ populations of Trans&GC B cells, naïve B cells, MZB cells, cMBCs and aMBCs from the spleen of HC (*n* = 12), HBV-LC (*n* = 16), and nonHBV-LC (*n* = 11) subjects as indicated. **e** Representative CD11c, CXCR3, and CCR6 expression profiles of the spleen B cell subsets from the spleens of one healthy donor and one HBV-LC donor. **f** Percentages of CD11c^+^ and CXCR3^+^ populations of the B cell subsets of subjects as in **c**, **d**. **g** Mean fluorescence intensity (MFI) of CCR6 on the indicated B cell subsets from the spleens of HC (*n* = 9), HBV-LC (12) and nonHBV-LC (*n* = 11) subjects. Data are shown as the mean and sem. *P < 0.05, **P < 0.01, ***P < 0.001, and ****P < 0.0001, as determined by Mann–Whitney *U*-test.

The persistent activation of splenic Tfh cells may enhance B cell maturation in cirrhotic patients^26^; therefore, we analyzed the activation-associated markers (CD95, FcRL4, CD86, and CD71) and migration-associated markers (CD11c, CXCR3, and CCR6) on the B cell subsets in LC patients (Table 2, panels 2 and 3). In these two panels, Trans&GC B cells, PBs, naïve B cells, and MZB cells were gated according to their phenotypes, while IgD^−^ memory B cells within CD38^+/−^CD10^−^ mature B cells were divided into CD27^+^ classical memory B cells (named cMBCs here) and CD27^−^ atypical memory B cells (named aMBCs here)^33^ (Figs. 1c and 2a). The expression of the activation-associated and migration-associated markers on the B cell subsets was then analyzed by gating the positive populations or by measuring the mean fluorescence intensity (MFI) (Fig. 2b, c). Overall, regardless of whether the B cells were harvested from the periphery or the spleen, the B cell subsets from LC patients, to varying extents, expressed higher levels of CD95, FcRL4, CD86, CD71, CD11c, CXCR3, and CCR6 than those from HC subjects (Fig. S2A–D and Fig. 2d–g). In particular, CD95, a death receptor that is highly expressed on apoptotic cells and activated cells, was significantly upregulated on nearly all the splenic B cell subsets in LC patients (Fig. 2d). Similarly, the inhibitory receptor FcRL4 was also found to be significantly increased on the splenic MZB cells in LC patients (Fig. 2d). FcRL4 is known to be a receptor for commensal microbial antigens^34^. The increase in FcRL4^+^ MZB cells may reflect the bacterial antigen accumulation or bacterial translocation in the spleens of LC patients. Interestingly, both CD86 and CD71 were found to be significantly increased on splenic Trans&GC B cells from LC patients, while their patterns were different in the periphery (Fig. 2e and Fig. S2B). As CD71 is also known to be a marker of pro-GC B cells^35^, this data suggests that splenic GC activity is enhanced during cirrhosis. Additionally, the upregulation of CD71 by peripheral B cells in CHB patients (Fig. S2B) was consistent with our previous findings^36^. CD11c, a marker for aMBCs that identifies an expanded B cell subset in various chronic inflammatory conditions^37^, was also increased in the splenic cMBCs, MZB cells, and aMBCs from nonHBV-LC patients (Fig. 2f). In addition, the splenic cMBCs and aMBCs from LC patients had higher levels of CXCR3 than those from HC subjects (Fig. 2f). Finally, compared with that in HC subjects, CCR6 expression was increased across multiple splenic B cell subsets in LC patients (Fig. 2g). Together, these results suggest a persistent activation of B cell compartments in cirrhotic patients, especially in the splenic B cell subsets.

### The enhanced GC response and decreased primary follicles in the spleens of cirrhotic patients

**Fig. 3:**
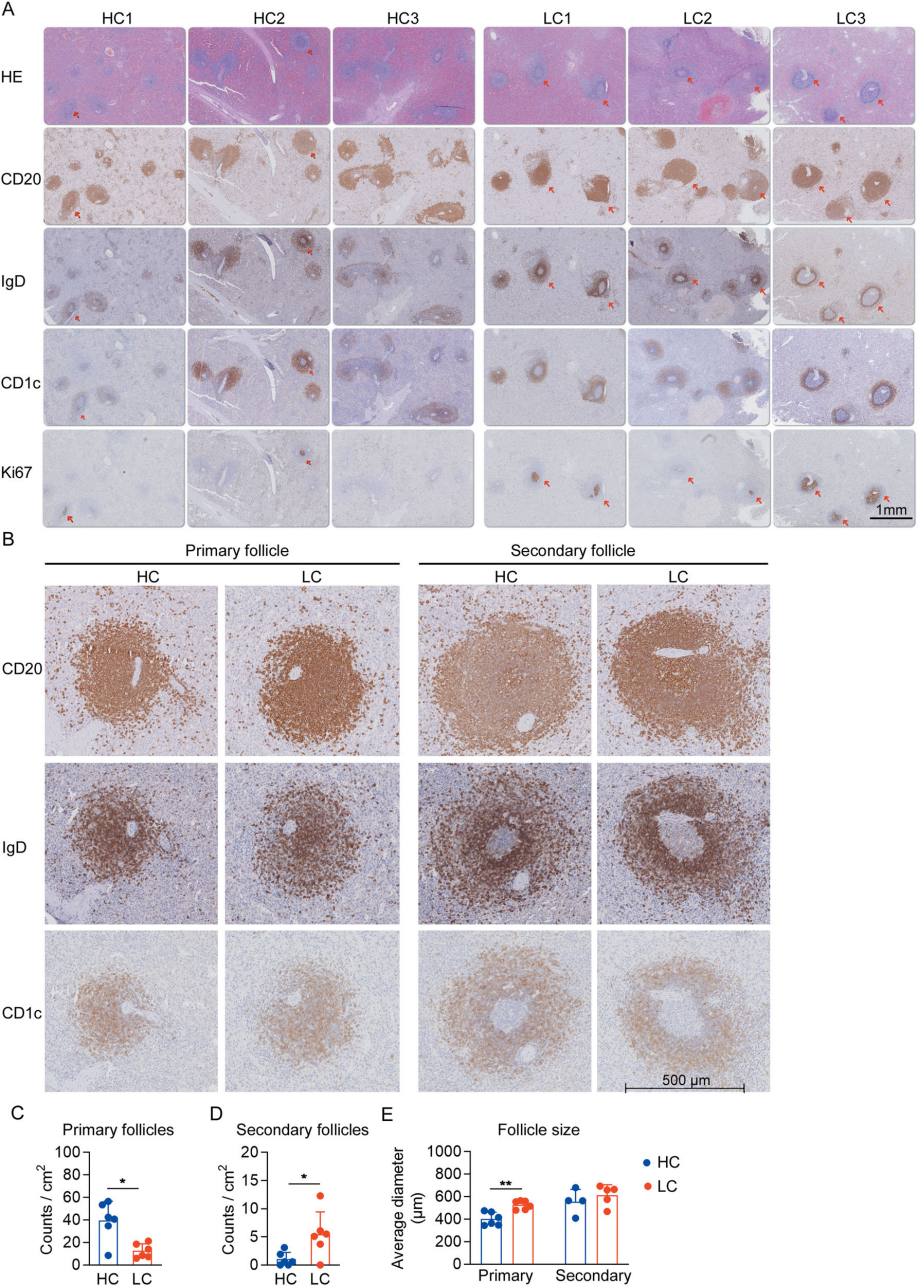
Immunochemistry staining showing the enhanced GC responses and decreased MZB-enriched primary follicles in LC patients. Formalin-fixed, paraffin-embedded splenic sections from healthy donors (*n* = 6) and LC patients (*n* = 6) were used for hematoxylin-eosin (HE) and immunohistochemical staining. **a** HE and CD20, IgD, CD1c and Ki-67 staining in serial/or adjacent splenic sections from three HC and three LC subjects. **b** Magnified representative primary and secondary follicles from HC and LC subjects stained with CD20, IgD, CD1c. **c**, **d** Average counts of the primary and secondary B cell follicles from HC (*n* = 6) and LC (*n* = 6) subjects. **e** Average diameter of the primary follicles from HC and LC subjects. Note that the secondary follicles could not be found in the sections from two HC and one LC subjects. *P < 0.05, and **P < 0.01, as determined by Mann–Whitney *U*-test.

As stated above, LC patients have a significant increase of Trans&GC B cells and loss of MZB cells and IgD^−^ memory cells in their spleens. To further investigate these changes in situ, we conducted immunohistochemistry staining and examined serial splenic sections with anti-CD20, anti-IgD, anti-Ki-67, and anti-CD1c, which were used to identify B cell follicles, splenic marginal zones, germinal centers and MZB cells^30,38,39^. We found that the spleens of cirrhotic patients generally showed enlarged GC-like secondary follicles (Fig. 3a). It is also obvious that IgD^hi^ naïve B cells (CD1c^−^) aggregated at the mantle zone in the spleen, forming a distinct ring-shaped layer surrounding the follicle center (Fig. 3a, b). Further staining of the B cell follicles with Ki-67 indicated increased proliferation in the pseudo-GC area in LC patients (Fig. 3a). While primary follicles showed comparable CD1c^+^ cell enrichment (mainly MZB cells) in both HC and LC subjects, the enlarged secondary follicles in LC patients exhibited a relative thinner marginal zone than those in HC subjects (Fig. 3a, b). Moreover, LC patients obviously had a much lower number of primary B cell follicles, which was accompanied by increased secondary follicle counts (Fig. 3c, d). Interestingly, the primary follicles of LC patients were larger than those of HC subjects (Fig. 3e), likely reflecting a more active state. Those increased secondary structures are consistent with increased Trans&GC B cells, while the lack of primary structure accompanies MZB cell loss in LC spleens.

### Bulk RNA-seq revealed metabolic defects of the splenic B cells in LC patients

**Fig. 4:**
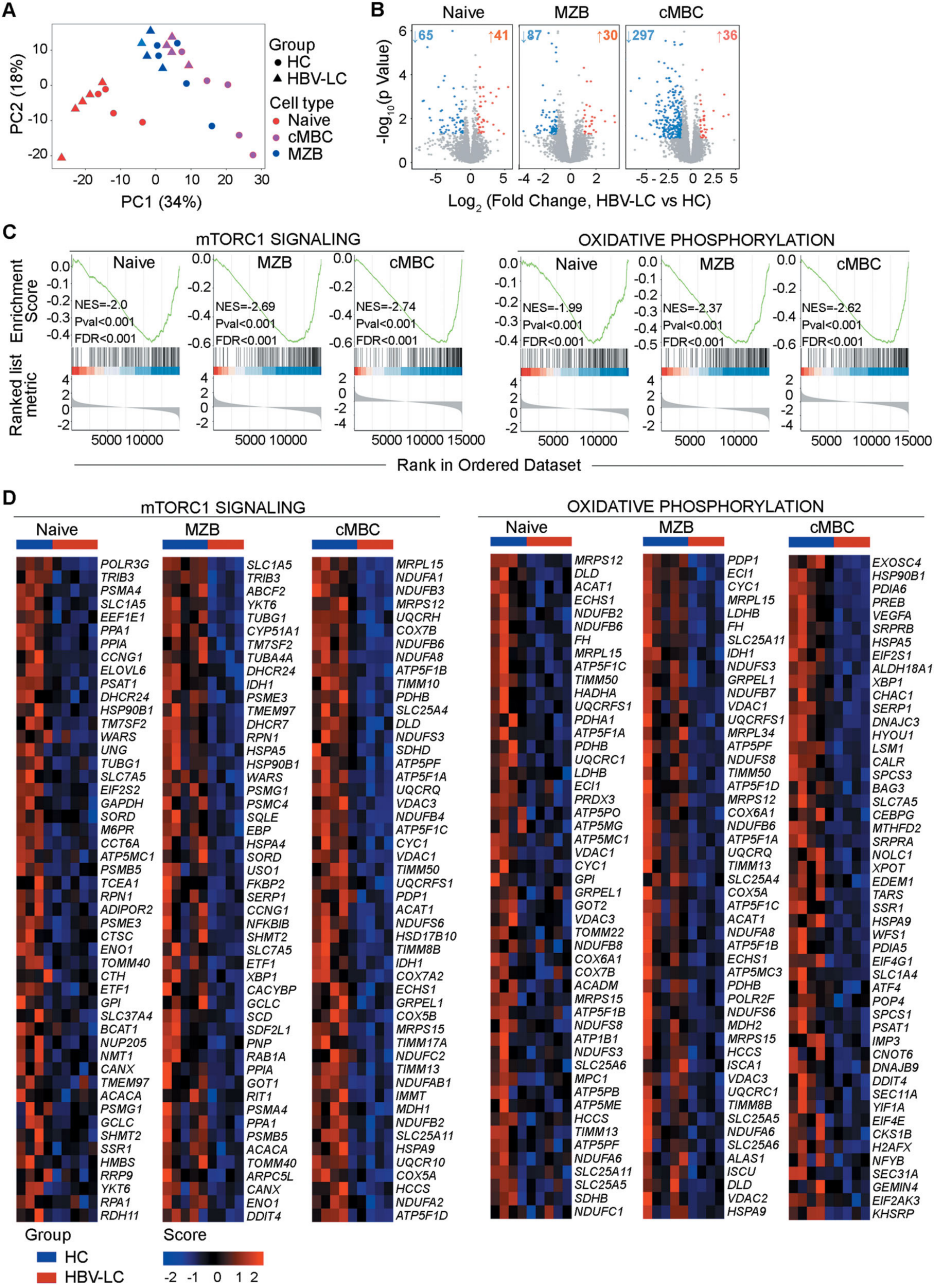
Transcriptomic comparisons of the splenic B cell subsets in healthy donors versus LC patients. **a** PCA plot shows the transcriptomes of naïve B cells, MZB cells, and cMBCs from HC and HBV-LC subjects. Each point represents one sample. **b** Volcano plots show the differentially expressed genes (DEGs) in the naïve B cells, MZB cells and cMBCs between HC and HBV-LC subjects. Each point represents one gene, and genes that had a log2(foldchange) < −1 or >1 and *P* value < 0.05 as measured by Wald test were identified as downregulated or upregulated genes, respectively. The numbers of downregulated or upregulated genes are indicated. **c** GSEA plots show the enriched gene sets in the mTORC1 signaling, oxidative phosphorylation pathways, which were significantly downregulated in the naïve B cells, MZB cells, and cMBCs of HBV-LC subjects. The *X*-axis of the green curve is the rank of the genes that were ranked according to signal-to-noise values, and the *Y*-axis is the enrichment score (ES). The black bars indicate the rank of the genes in certain pathways. The genes on the right side of the peak indicate the leading edge subset genes that contribute the most to the ES. **d** Heatmaps show the genes (Top 50 arranged by signal to noise, see “Materials and methods” section) involved in the mTORC1 signaling and oxidative phosphorylation pathways that were downregulated in the naïve B, MZBs, and cMBCs from HBV-LC subjects compared with those from HC subjects.

To further explore the overall impact of liver cirrhosis on B cell subsets in LC patients, we performed bulk RNA-seq on the sorted splenic naïve B cells, MZB cells and cMBCs from HBV-LC and HC subjects. We profiled the transcriptome of the B cell subsets through principal component analysis (Fig. 4a). It is shown that the samples were clustered by B cell subsets rather than by subject groups. The transcriptomes of the MZB cells and cMBCs were more similar to each other but distinct from that of the naïve B cells. There were 41 upregulated and 65 downregulated genes observed in the naïve B cells, 30 upregulated and 87 downregulated genes observed in the MZB cells and 36 upregulated and 297 downregulated genes observed in the cMBCs in LC patients compared to those in HC subjects (Fig. 4b).

Gene set enrichment analysis (GSEA) was used to identify the possible functional alterations in the B cell subsets from LC patients. Based on the hallmark gene sets in MSigDB, we confirmed that the mTORC1 signaling and oxidative phosphorylation pathways were significantly downregulated in all three B cell subsets from LC patients (Fig. 4c). The RNA-seq data also revealed a defect in glycolysis in splenic naïve B cells, MZBs and cMBCs of LC patients (Fig. S3A). Indeed, the expression levels of multiple genes in mTORC1 signaling, oxidative phosphorylation and glycolysis pathways were decreased (Fig. 4d and Fig. S3B). These results strongly suggest a broad metabolic defect of splenic B cells in LC patients. Furthermore, genes involved in the negative regulation of cell apoptosis and the positive regulation of cell proliferation were also downregulated in cMBCs and MZB cells in LC patients (Fig. S3C). Thus, we speculated that in cirrhosis, splenic B cells exhibit profound functional impairments in metabolism, antiapoptotic pathways, proliferation and/or differentiation.

### The functional impairment of B cell compartments from cirrhotic patients

**Fig. 5:**
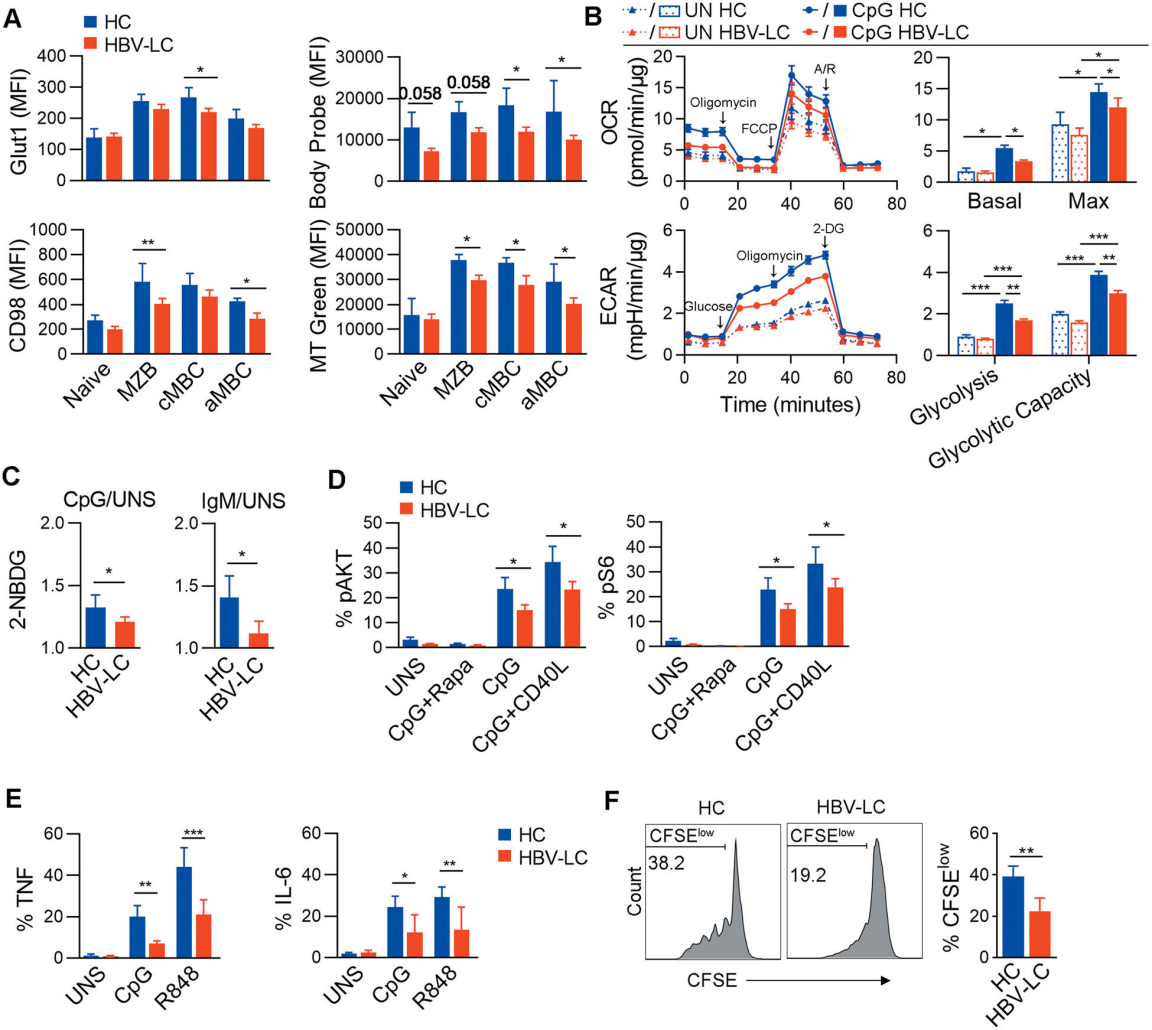
B cells from LC patients show defective metabolism and impaired function. **a** Pooled data indicated the expression of metabolic markers, including GLUT1, CD98, BodyProbe, and MitoTracker Green (MT green) by the splenic B cell subsets from HC (*n* = 4) and HBV-LC (*n* = 4) subjects. **b** The oxygen consumption rate (OCR) and extracellular acidification rate (ECAR) were measured in vitro in the enriched splenic B cells from HC (*n* = 3) and HBV-LC (*n* = 3) subjects using a Seahorse analyzer. The cells were either untreated or stimulated with CpG. Oligomycin, carbonyl cyanide-4-(trifluoromethoxy) phenylhydrazone (FCCP, mitochondrial uncoupling agent), antimycin A, rotenone (A/R) and 2-deoxy-glucose (2-DG) were added at the indicated times. **c** The uptake of 2-(N-(7-nitrobenz-2-oxa-1,3-diazol-4-yl)amino)-2-deoxyglucose (2-NBDG) by the splenic B cells from HBV-LC and HC subjects cultured in vitro with or without CpG (HC, *n* = 6; HBV-LC, *n* = 5) or IgM (HC, *n* = 4; HBV-LC, *n* = 4) for 24 h. **d** pAKT (Thr308) and pS6 (Ser235/236) levels of splenic B cells from HC (*n* = 3) and HBV-LC (*n* = 4) subjects, either unstimulated (UNS) or stimulated with CpG (1 μM), CpG (1 μM) plus rapamycin (Rapa, 10 nM) or CpG (1 μM) plus CD40L (500 ng/mL). **e** Cytokine (TNF and IL-6) production by the splenic B cells from HC (*n* = 4) and HBV-LC (*n* = 4) subjects cultured in vitro unstimulated or stimulated with CpG (1 μM) or R848 (1 μg/mL) for 18 h, at the present of brefeldin A. **f** Proliferation of the splenic B cells from HC (*n* = 4) and HBV-LC (*n* = 4) subjects cultured in vitro with CpG for 4 days. CFSE was used to label the enriched splenic B cells, and CFSE^low^ proliferating cells were measured. UNS unstimulated. Data are shown as the mean and SD. *P < 0.05, and **P < 0.01, as determined by Mann–Whitney *U*-test.

We further confirmed the defective metabolism of splenic B cells in LC patients. Glucose is dispensable for B cell activation, and oxidative phosphorylation is also fueled by other nutrients^40^. We found that the levels of glucose transporter GLUT1 on cMBC subset and the expression of CD98 (SLC3A2), a heavy chain of the heterodimeric amino acid transporter that was associated with proliferating cells^41^, by the MZB cell and aMBC subsets were significantly reduced in LC patients (Fig. 5a). In addition, CD36 (fatty-acid transporter) expression by all three B cell subsets was similar between LC patients and HC subjects (Fig. S4). However, we did observe significantly decreased uptake of fatty acids in naïve B cells, MZB cells, and cMBCs in LC patients (Fig. 5a). Mitochondrial dynamics play an important role in energy production of immune cells^42^. We also measured the splenic B cell mitochondrial mass to assess metabolic activity^43^ using MitoTracker Green^44^ and observed a significant decrease in the cMBCs, MZB cells, and aMBCs of LC patients compared to that in HC subjects (Fig. 5a). Moreover, we used Seahorse to analyze B cell oxygen consumption rate (OCR) and the extracellular acidification rate (ECAR), which measure mitochondrial energy production and lactate production by glycolysis, respectively, and we found that cultured B cells from cirrhotic patients have profound defects in oxygen consumption and glycolytic function in both untreated and stimulated conditions (Fig. 5b). In particular, there was significantly lower maximal respiratory potentials in the B cells from cirrhotic patients when stimulated by CpG (Fig. 5b). A 24 h culture also showed a decreased uptake of 2-(N-(7-nitrobenz-2-oxa-1,3-diazol-4-yl)amino)-2-deoxyglucose (2-NBDG, a fluorescent glucose analog that is used to monitor glucose uptake in live cells) by splenic B cells from cirrhotic patients compared to those from healthy subjects (Fig. 5c). Together, these data indicated that liver cirrhosis likely caused profound impairment in utilizing amino acids and fatty acids for oxidative phosphorylation by the various splenic B cell subsets.

We next examined whether the B cells from cirrhotic patients exhibit functional impairments in mTORC1-related signaling transduction, cytokine production and cell proliferation in response to stimulation. Indeed, we observed a significantly lower levels of pAKT and pS6 in HBV-LC splenic B cells stimulated by CpG or CpG/CD40L (Fig. 5d), confirming a defect in mTORC1 activation. We also found that the cytokine production of the splenic B cell subsets was much lower in HBV-LC patients than in HC subjects (Fig. 5e). In addition, the splenic B cells in HBV-LC patients displayed a much lower in vitro proliferation capacity after 4 days of culture than those in HC subjects (Fig. 5f). Thus, our data reveal that B cells from LC patients are hyperactive and functionally impaired and exhibit poor proliferative capacity, which are associated with their defective energy metabolism.

## Discussion

Little is known regarding how liver cirrhosis affects splenic B cells. Here, we described the disturbed splenic B cell compartments and their hyperactivation phenotypes in LC patients. Our data also suggest that metabolic defects are the potential mechanisms for the splenic B cell functional impairment in LC patients. These defects in the B cell subsets may be linked to the diminished humoral immunity in cirrhotic patients.

The dysregulated B cell compartments are unique in cirrhotic patients and are different from the B cell compartments previously reported in other chronic inflammatory conditions, such as HIV, HCV, malaria, and autoimmunity^16–21^. Although the CD27^+^ memory B cells are significantly depleted, liver cirrhosis does not promote the expansion of CD11c^+^Tbet^+^ aMBCs but significantly increases the naïve B cell and Trans&GC B cell populations. In addition, CD21 expression in the IgD^+/−^/CD27^+^ B cell subsets was largely maintained in LC, which differs from other conditions^16,20,21^. The perturbations of splenic B cell compartments are not specific to HBV infection, since the B cell compartments in nonHBV cirrhosis also show similar perturbation as evident by the flowcytometry data. Together with other reports^13^, our data suggest a nonHBV-dependent mechanism underlying the dysregulation of B cell compartments during liver cirrhosis. Although it is possible that persistent HBV antigens may nonspecifically affect B cells^45–47^, we did not observe significant changes in the mature B cell compartments from the CHB patients enrolled in the current study. Future studies should examine whether HBV-associated antigens affect B cell properties in larger patient cohorts with different clinical phases.

Persistent activation of spleen B cells are evident in LC patients, However, the mechanisms involved in memory B cell loss and functional impairment are not well understood. Here, we showed that defective metabolism is likely one of such missing links. Hyperinflammatory cues in LC patients may rewire the metabolic regulatory network. The bulk transcriptomic data reveal the broad defects in the cellular energy metabolic pathways in both the switched and nonswitched memory B cell subsets from LC patients; these data were subsequently confirmed by in vitro functional assays, which showed the impaired metabolic capacity and proliferation of the B cells. These phenomena are similar to the immune paralysis status reported in sepsis patients^48^, in which both glycolysis and oxidative metabolism are defective. In addition, the preferential loss of MZB cells is likely due to the increased activation and differentiation of B cells into plasma cells during LC. A previous study showed that IgM^+^CD27^+^ B cells have a higher capacity to migrate toward CXCL13^49^, suggesting an ability of these cells to enter B cell follicles. Indeed, we observed a higher expression of CXCL13 in cirrhotic patients, and the increased splenic Tfh cells may facilitate the differentiation of plasma cells^26^. These data suggested that the increased engagement of MZB cells in GC reactions likely exhausts the splenic MZB cell pools during liver cirrhosis. Another possibility is increased activation-induced cell death, which is associated with the loss of CD27^+^ B cells^50^. Our transcriptomic analysis showed perturbation of the regulatory network related to B cell death and survival in LC patients. Indeed, we observed the defective proliferative capacity of LC B cells. This did not contradict with the increased splenic ki-67 immunochemistry staining which concentrated in the pseudo-GC area and indicated mostly GC B cells. Thus, through the comprehensive analysis of the splenic B cell subsets from LC patients, we proposed that defective and impaired metabolic capacities and heightened levels of differentiation and activation potentially exhaust the memory B cell pool and functionality.

SLOs are the sites where B cells respond to antigens. The spleen is the largest SLO and contains numerous B cells, particularly MZB cells^23,24^. Although the depletion of peripheral memory B cells during liver cirrhosis has been reported^3,13^, little is known regarding the depletion of memory B cells in the SLOs. The splenic histology shows profound changes in B cell distributions along with increased naïve B and decreased memory B cells in patients. It is interesting to speculate that the distinct naïve B cell rings closely surrounding the GCs may affect the humoral immune response. More importantly, we identified a series of cirrhosis-associated pathways in splenic B cells for future characterization.

In summary, liver cirrhosis profoundly impacts B cell compartments, which may contribute to defective humoral immunity. A better understanding of this process may help to design intervention strategies to reinvigorate B cell functions.

## Materials and methods

### Enrolled subjects and sample processing

Cirrhotic spleen samples were collected from 44 hepatitis B virus-associated cirrhosis (HBV-LC) subjects and 11 non-hepatitis B virus-associated cirrhosis (nonHBV-LC) subjects who underwent splenectomy to relieve the symptoms caused by portal hypertension and splenomegaly. Healthy spleen samples were collected from 27 liver transplantation donors as controls. Blood samples were taken from 24 HBV-LC, 16 nonHBV-LC, 34 chronic hepatitis B (CHB) patients and 27 age-matched healthy donors who had no evidence of infection, liver diseases, or autoimmune diseases. All patients and heathy donors were recruited from Shenzhen 3rd People’s Hospital. Informed consent was obtained from all the participants. The protocol was approved by the ethics committee of Shenzhen 3rd People’s Hospital (reference 2020-130). The basic demographic information of the enrolled subjects is listed in Table 1.

Spleen cells and PBMCs were isolated according to our previously published protocols^26^. For FACS staining, the cells were either used fresh or were cryopreserved. For bulk RNA sequencing, fresh cells were used. For the cell culture experiments, cryopreserved samples were used.

### Antibodies and flow cytometry

The detailed information about the antibodies used to analyze the B cell subsets, phenotypes, and functions are listed in Supplementary Table 1. Fatty acid uptake was tested by BODIPY™ FL C16 (Invitrogen, Cat. D3821). To identify and exclude the dead cells, aqua fluorescent reactive dye (Invitrogen, Cat. L34957) was used according to the manufacturer’s instructions. For surface marker staining, the cells were incubated with antibodies on ice for 30 min and then washed and fixed for further analysis. Intracellular staining was performed using the relevant antibodies in Perm/Wash Buffer (BD Biosciences) or the Foxp3/Transcription Factor Staining Buffer Set (eBioscience^TM^, Cat. 00-5523). The samples were analyzed for B cell markers by FACS Frotessa and for metabolic markers by FACSCanto II flow cytometer (BD Biosciences), and the data were further analyzed by FlowJo software v.10 (TreeStar).

### Immunohistochemistry

Hematoxylin-eosin (HE) and immunohistochemistry staining were performed using paraffin-embedded 4-µm splenic tissue serial sections. The information about the primary mAbs is listed in Supplementary Table 1. The sections were first incubated with the optimal concentrations of the primary antibodies for 1 h at room temperature (RT). Then, the sections were incubated with the HRP-goat-rabbit/mouse antibodies (Cat. PV-6000, ZS-GB BIO) and were subsequently developed by DAB (Cat. ZLI-9018, ZS-GB BIO). All the slides were visualized using the NanoZoomer-XR Digital slide scanner C12000 under the 0.23 μm/pixel mode, and the images were analyzed by NDP.view2 software. B cell follicles were determined by CD20 staining, with identification of primary follicles by no detection of germinal center formation, while the secondary follicles processed obvious germinal centers which were negative of IgD and positive of Ki-67^30,38,39^.

### Metabolic analysis

MitoTracker Green (Invitrogen, Cat. M7541) was used to examine the mitochondrial mass. For MitoTracker Green staining and fatty acid uptake, splenic cells or magnetic bead-enriched B cells were resuspended in a 1/1000 dilution of Fixable Viability Dye eFluor® 450 (Thermo Fisher). Then, the cells were incubated at room temperature for 15 min, washed and resuspended with prewarmed, freshly made MitoTracker Green staining buffer (50 µM) or BODIPY™ FL C16 staining buffer (2 µM) at 37 °C for an additional 30 min. The cells were centrifuged, resuspended and stained with the other antibodies. The B cell oxygen consumption rate (OCR) and extracellular acidification rate (ECAR) were determined by a Seahorse XF platform according to the manufacturer’s instructions (Agilent Technologies). 2-NBDG (Thermo Fisher, Cat. N13195) uptake was performed as described^51^.

### mTOR1 signaling and cytokine production assay

For assays examining pAKT and pS6 levels, enriched splenic B cells (1 × 10^6^ cells/mL) were cultured unstimulated or stimulated with CpG ODN2006 (1 μM), CpG (1 μM) plus rapamycin (Rapa, 10 nM) or CpG (1 μM) plus CD40L (500 ng/mL) for 10 h, then pAKT (Thr308) and pS6 (Ser235/236) were analyzed by intracellular staining with PE anti-pAKT and AF488 anti-pS6. For cytokine production assay, enriched splenic B cells were stimulated by CpG (1 μM) or medium with BFA (1 μg/mL) for 18 h in 96-well U-bottom plates (Costar, Cat. 3799). Then, the cells were collected for surface marker staining, followed by intracellular staining for TNF and IL-6, and analyzed by flow cytometry.

### In vitro B cell proliferation and differentiation assay

The enriched splenic B cells were labeled with CFSE and cultured in 96-well U-bottom plates with or without CpG (1 μM) at a concentration of 1 × 10^6^ cells/mL for 4 days. Then, the proliferating cells (CFSE^low^) were analyzed by flow cytometry.

### Sample processing for bulk RNA-seq

For bulk RNA-seq, freshly isolated B cells were enriched using a B Cell Isolation Kit II (Miltenyi Biotec, Cat. 130-091-151). Then, the enriched B cells were stained with anti-CD19 PerCP-Cy5, anti-CD10 PE, anti-CD27 PE-Cy7, anti-IgD PE-CF594, and anti-IgM APC for 30 min, washed and resuspended at a concentration of 10^7^ cells/mL. A FACS Aria II cell sorter was used to sort CD19^+^CD10^−^IgD^+^IgM^+^CD27^+^ MZB cells, CD19^+^CD10^−^IgD^+^IgM^+^CD27^−^ naïve B cells, and CD19^+^CD10^−^IgD^−^IgM^−^CD27^+^ cMBCs. At least 1 × 10^6^ cells were collected for each sample. The collected B cell subsets were centrifuged after a purity check on the sorter, and the subsets were suspended in TRIzol and stored at −80 °C until RNA extraction. A total of 27 samples from five HC subjects and five HBV-LC patients were ultimately collected for bulk RNA-seq.

### Bulk RNA-seq mapping and GSEA

The samples were sequenced on an Illumina HiSeq X Ten platform. Paired-end libraries were constructed with an insert size of 250–300 bp. The length of each read was 150 bp. The clean reads were mapped to the human genome (hg38) by applying HISAT2^52^ with the default parameter. Then, we calculated the gene count using feature counts of SUBREAD^53^ with default parameters. The genes with fewer than ten gene counts were discarded. Then, the gene abundances were compared using the R package of DESeq2^54^. PCA was also performed by DESeq2 with rlog normalized gene abundance. Differentially expressed genes (DEGs) were calculated by Wald test, by which genes that had a log_2_(foldchange) of < −1 or > 1(*p* value < 0.05) were identified as downregulated or upregulated genes, respectively. GO analysis was performed in DAVID (https://david.ncifcrf.gov/) using the differentially expressed genes. The GSEA was performed using the R package of clusterProfiler (Version 3.12.0)^55^, in which the hallmark gene sets in MSigDB^56^ were used for annotation. Genes for GSEA were ranked by signal to noise ratio, with the formula *μ*_A_ − *μ*_B_/*σ*_A_ − *σ*_B_, where *μ* is the mean and *σ* is the standard deviation; *σ* has a minimum value of 0.2.

### Statistics

All the statistical analysis was performed using GraphPad Prism Version 8.0.2. The data are presented as the mean values with SDs or SEMs. Multiple comparisons among the different groups were first performed using the nonparametric Kruskal–Wallis *H*-test. Comparisons between various groups were made using the Mann–Whitney *U*-test, whereas comparisons between the same individual were made using Wilcoxon’s matched-pairs test. Correlations between two variables were analyzed using the Spearman rank correlation test. *P* < 0.05 on two sides was considered to be significant for all the analyses.

## Supplementary information

Supplementary Figure Legends

Supplementary Tables

Supplementary Figure 1

Supplementary Figure 2

Supplementary Figure 3

Supplementary Figure 4
