# Supplementary material for: Metabolic defects in splenic B cell compartments from patients with liver cirrhosis
Source: Cell Death Dis. 2020 Oct 24;11(10):915. doi: 10.1038/s41419-020-03060-1 (PMC7585577; doi:10.1038/s41419-020-03060-1)
Supplement: Supplementary file 1 — Supplementary Figure Legends [file 41419_2020_3060_MOESM1_ESM.docx]

**Supplementary Figure Legends**

**Supplementary Figure 1. (A)** The frequencies of CD19^+^ B cells among CD45^+^ cells from the PBMCs of HC (n = 16), CHB (n = 26), HBV-LC (n = 12) and non-HBV-LC (n = 15) subjects and from the spleens of HC (n = 14), HBV-LC (n = 22) and non-HBV-LC (n = 11) subjects. The spleens have obviously higher B cell percentages than the PBMCs, independent of disease status. (**B**) The MZB cell subset frequencies in the spleens from HBV-LC patients with different Child-Pugh scores. (**C**) Correlation of the spleen MZB frequency with the white blood cell counts and platelet counts in HBV-LC patients. No significant correlation was found.

**Supplementary Figure 2. Phenotypical analysis indicates the persistent activation of the peripheral B cell compartments in LC patients.** (**A**) and (**B**) Percentages of the CD95+, FcRL4+, CD86+ and CD71+ populations of Trans&GC B cells, naïve B cells, MZB cells, cMBCs and aMBCs from the peripheral B cells of HC, HBV-LC, non-HBV-LC and CHB subjects as indicated. (**C**) Percentages of CD11c+ and CXCR3+ populations of the peripheral B cell subsets of subjects as in (A) and (B). (**D**) Mean fluorescence intensity (MFI) of CCR6 on the indicated B cell subsets from the peripheral of HC and HBV-LC subjects. Data are shown as the mean and sem. *P < 0.05, **P < 0.01, ***P < 0.001, and ****P < 0.0001, as determined by Mann-Whitney U test. The number of study subjects are shown in supplementary table 2 (**Table S2**).

**Supplementary Figure 3. Transcriptomic comparisons of the splenic B cell subsets in healthy donors versus LC patients.** (**A**) GSEA plots show the enriched gene sets in glycolysis, which were significantly downregulated in the naïve B cells, MZB cells and cMBCs of HBV-LC subjects. The X-axis of the green curve is the rank of the genes that were ranked according to signal-to-noise values, and the Y-axis is the enrichment score (ES). The black bars indicate the rank of the genes in certain pathways. The genes on the right side of the peak indicate the leading edge subset genes that contribute the most to the ES. (**B**) Heatmaps show the genes (Top 50 arranged by signal to noise, see Materials and Methods) involved in the glycolysis that were downregulated in the naïve B, MZBs and cMBCs from HBV-LC subjects compared with those from HC subjects. (**C**) Heatmaps showing the genes involved in the negative regulation of apoptosis and the positive regulation of proliferation that were downregulated in the splenic cMBCs and MZB cells from LC patients based on Gene Ontology (GO) analysis.

**Supplementary Figure 4. CD36 expression levels in the splenic B cell subsets were comparable between cirrhotic patients and healthy controls.** Pooled data indicate the expression of CD36 in the splenic B cell subsets from HC (n = 4) and HBV-LC (n = 3) subjects as analyzed by FACS cytometry.
