# Supplementary material for: Metabolic defects in splenic B cell compartments from patients with liver cirrhosis
Source: Cell Death Dis. 2020 Oct 24;11(10):915. doi: 10.1038/s41419-020-03060-1 (PMC7585577; doi:10.1038/s41419-020-03060-1)
Supplement: Supplementary file 2 — Supplementary Tables [file 41419_2020_3060_MOESM2_ESM.docx]

**Supplementary Tables**

**Table S1. Antibody list for flow cytometry and immunohistochemistry**

| Marker | Fluorochrome | Clone | Cat. Number | Manufacture |
| --- | --- | --- | --- | --- |
| CD45 | APC-H7 | 2D1 | 560178 | BD |
| CD19 | BUV496 | SJ25C1 | 564655 | BD |
| CD19 | BUV395 | SJ25C1 | 563549 | BD |
| CD19 | BV711 | SJ25C1 | 563036 | BD |
| CD10 | BV605 | HI10a | 312222 | Biolegend |
| CD10 | PE | HI10a | 312203 | Biolegend |
| CD21 | PE-Cy7 | B-ly4 | 561374 | BD |
| CD21 | PE-CF594 | B-ly4 | 563474 | BD |
| CD27 | PE-Cy7 | M-T271 | 356412 | Biolegend |
| CD27 | BV785 | O323 | 302832 | Biolegend |
| CD38 | BV711 | HIT2 | 303528 | Biolegend |
| CD38 | PE-Cy7 | HB7 | 356608 | Biolegend |
| IgD | BV421 | IA6-2 | 348226 | Biolegend |
| IgD | PE-CF594 | IA6-2 | 562540 | BD |
| IgM | PE | G20-127 | 555783 | BD |
| IgM | BV650 | MHM-88 | 314526 | Biolegend |
| IgM | APC | MHM-88 | 314510 | biolegend |
| IgG | BUV395 | G18-145 | 564229 | BD |
| IgA | PerCPVio700 | IS118E10 | 130-107-053 | Miltenyi Biotec |
| IgG1 | PE | HP6001 | 9054-01 | Southern Biotech |
| IgG2 | AF647 | HP6002 | 9070-31 | Southern Biotech |
| IgG3 | AF488 | HP6050 | 9210-30 | SouthernBiotech |
| CD11 | APC | S-HCL-3 | 340544 | BD |
| CXCR3 | PcrCP-Cy5.5 | G025H7 | 353714 | Biolegend |
| CCR6 | PE | G034E3 | 353410 | Biolegend |
| FcRL4 | PerCP-eFluor 710 | 413D12 | 46-3079-42 | eBioscience |
| CD71 | BV711 | M-A712 | 563767 | BD |
| CD86 | BV650 | IT2.2 | 305428 | Biolegend |
| CD86 | BV711 | IT2.2 | 305440 | Biolegend |
| CD95 | PE | DX2 | 555674 | BD |
| IL-6 | PE | MQ2-13A5 | 501107 | Biolegend |
| TNF | APC | MAb11 | 502912 | Biolegend |
| CD98 | FITC | 5E5 | 11-0982-42 | Invitrogen |
| CD36 | FITC | eBioNL07 | 11-0369-41 | Invitrogen |
| GLUT1 | FITC | EPR3915 | ab195359 | Abcam |
| p-Akt (Thr308) | PE | D25E6 | 4854S | CST |
| p-S6 (Ser235/236) | AF488 | 2F9 | 13842S | CST |
| CD20 |  | rabbit polyclonal | PA5-16701 | Thermo Fisher Scientific |
| IgD |  | rabbit polyclonal | CAT ZA-0443 | ZS-GB BIO |
| CD1c |  | OTI1H9 | TA505384 | OriGene Technologies |
| Ki-67 |  | EP5 | ZA-0502 | ZS-GB BIO |

**Table S2. The subject numbers used for analysis of PBMC B cell activation and migration markers**

| Markers | HC | CHB | HBV-LC | Non-  HBV-LC |
| --- | --- | --- | --- | --- |
| CD95 | 8 | 7 | 11 | 12 |
| FcRL4 | 8 | 3 | 9 | 12 |
| CD86 | 16 | 26 | 7 | 12 |
| CD71 | 8 | 7 | 11 | 4 |
| CD11c | 17 | 30 | 11 | 16 |
| CXCR3 | 17 | 30 | 11 | 16 |
| CCR6 | 6 | - | 6 | - |

- : unstained or the data acquired are not in parallel for comparison.
