# Supplementary figures and images for: Metabolic defects in splenic B cell compartments from patients with liver cirrhosis
Source: Cell Death Dis. 2020 Oct 24;11(10):915. doi: 10.1038/s41419-020-03060-1 (PMC7585577; doi:10.1038/s41419-020-03060-1)

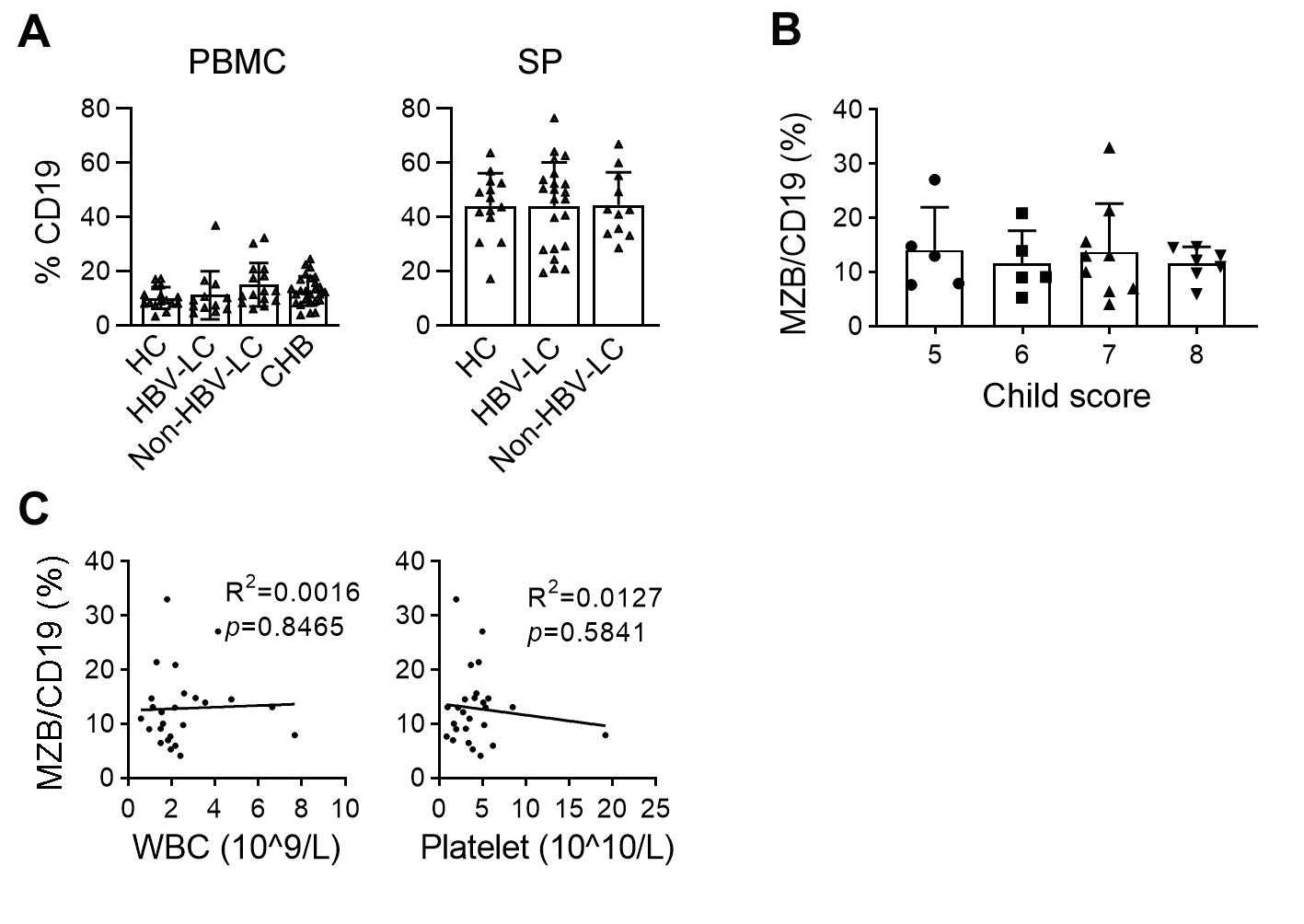

Supplement: Supplementary file 3 — Supplementary Figure 1 [file 41419_2020_3060_MOESM3_ESM.tif]

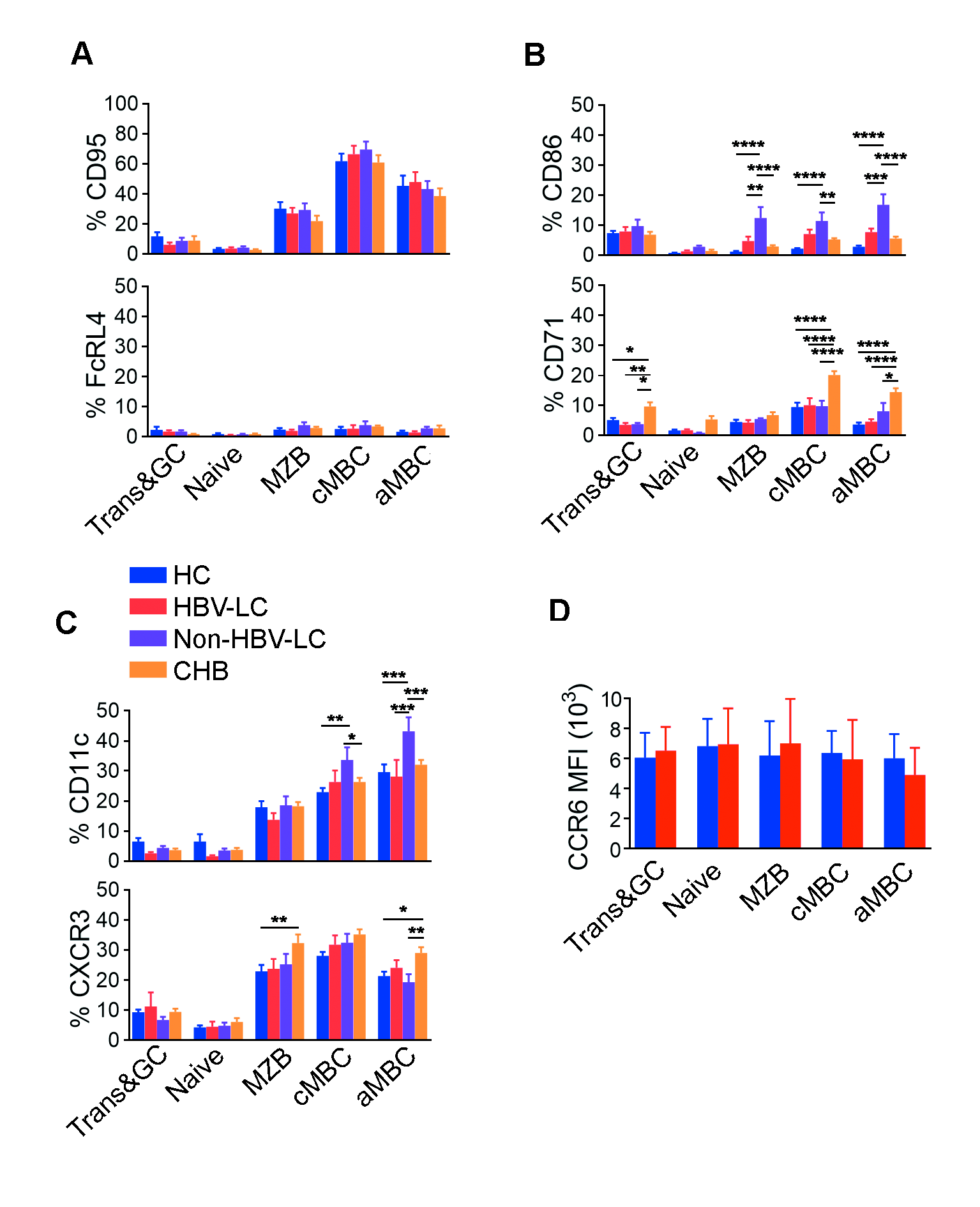

Supplement: Supplementary file 4 — Supplementary Figure 2 [file 41419_2020_3060_MOESM4_ESM.tif]

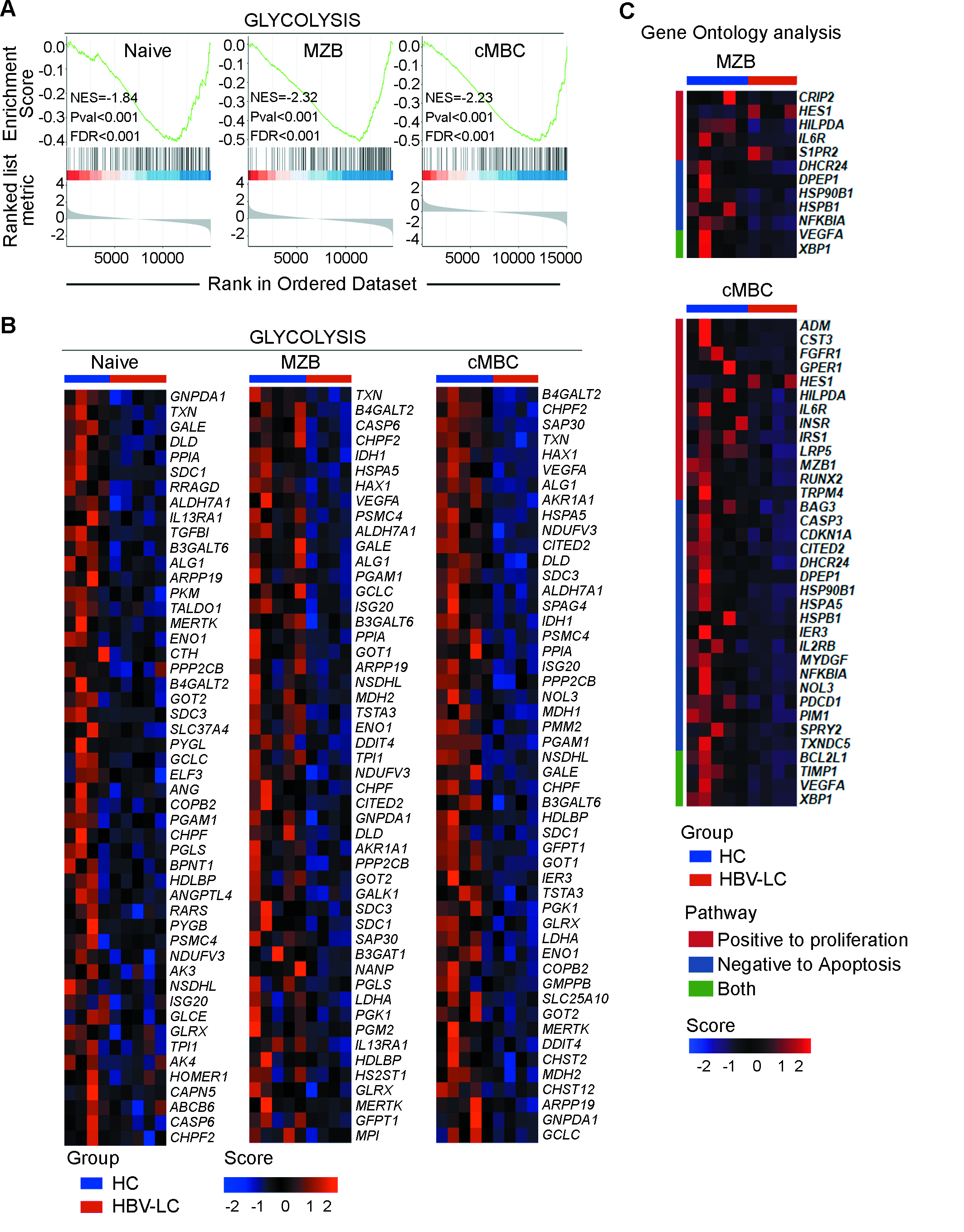

Supplement: Supplementary file 5 — Supplementary Figure 3 [file 41419_2020_3060_MOESM5_ESM.tif]

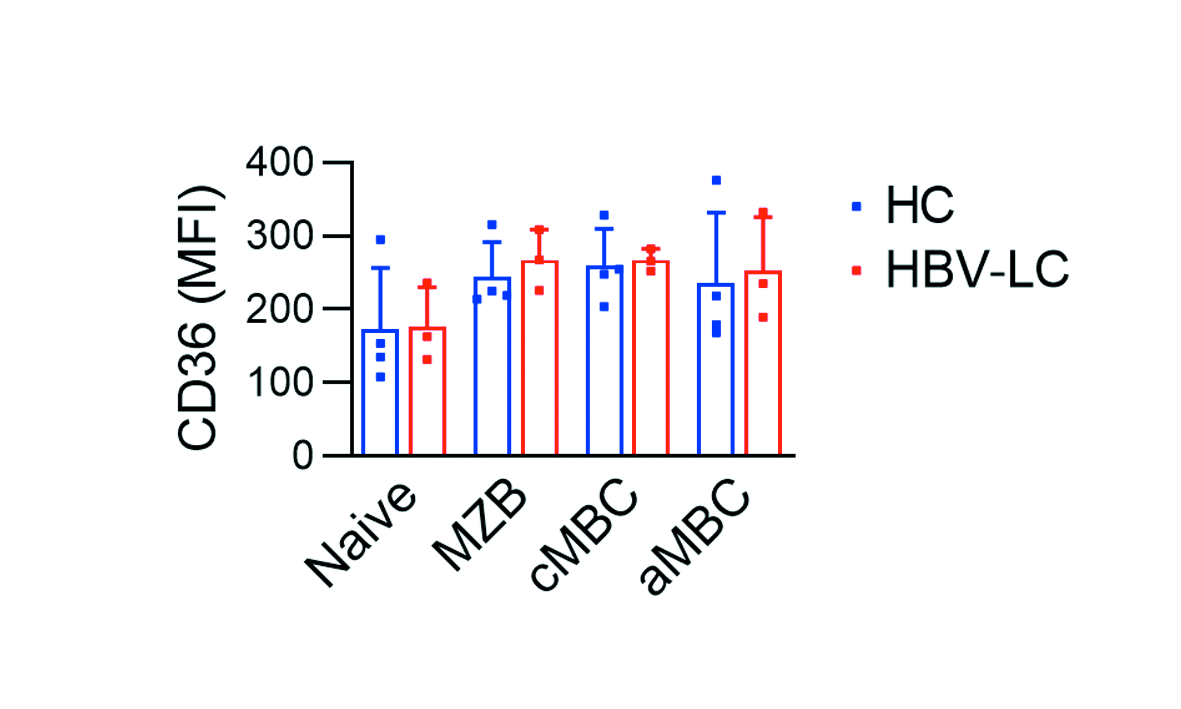

Supplement: Supplementary file 6 — Supplementary Figure 4 [file 41419_2020_3060_MOESM6_ESM.tif]
